# Recent advances in understanding the complexities of metastasis

**DOI:** 10.12688/f1000research.15064.2

**Published:** 2018-09-10

**Authors:** Jessica L. Chitty, Elysse C. Filipe, Morghan C. Lucas, David Herrmann, Thomas R. Cox, Paul Timpson

**Affiliations:** 1 Garvan Institute of Medical Research & the Kinghorn Cancer Centre, Cancer Division, Sydney, NSW, 2010, Australia; 2 St Vincent’s Clinical School, Faculty of Medicine, UNSW Sydney, NSW , 2010, Australia

**Keywords:** Metastasis, Cancer, Cancer Therapy, Extracellular Matrix, Tumour Stroma, Microenvironment, Intravital Imaging, Mouse Models, Biosensors, Circulating Tumour Cells, Disseminated Tumour Cells, Dormancy, Colonisation, Intravasation, Extravasation, Invasion, Migration

## Abstract

Tumour metastasis is a dynamic and systemic process. It is no longer seen as a tumour cell-autonomous program but as a multifaceted and complex series of events, which is influenced by the intrinsic cellular mutational burden of cancer cells and the numerous bidirectional interactions between malignant and non-malignant cells and fine-tuned by the various extrinsic cues of the extracellular matrix. In cancer biology, metastasis as a process is one of the most technically challenging aspects of cancer biology to study. As a result, new platforms and technologies are continually being developed to better understand this process. In this review, we discuss some of the recent advances in metastasis and how the information gleaned is re-shaping our understanding of metastatic dissemination.

## Introduction

**Figure 1.**
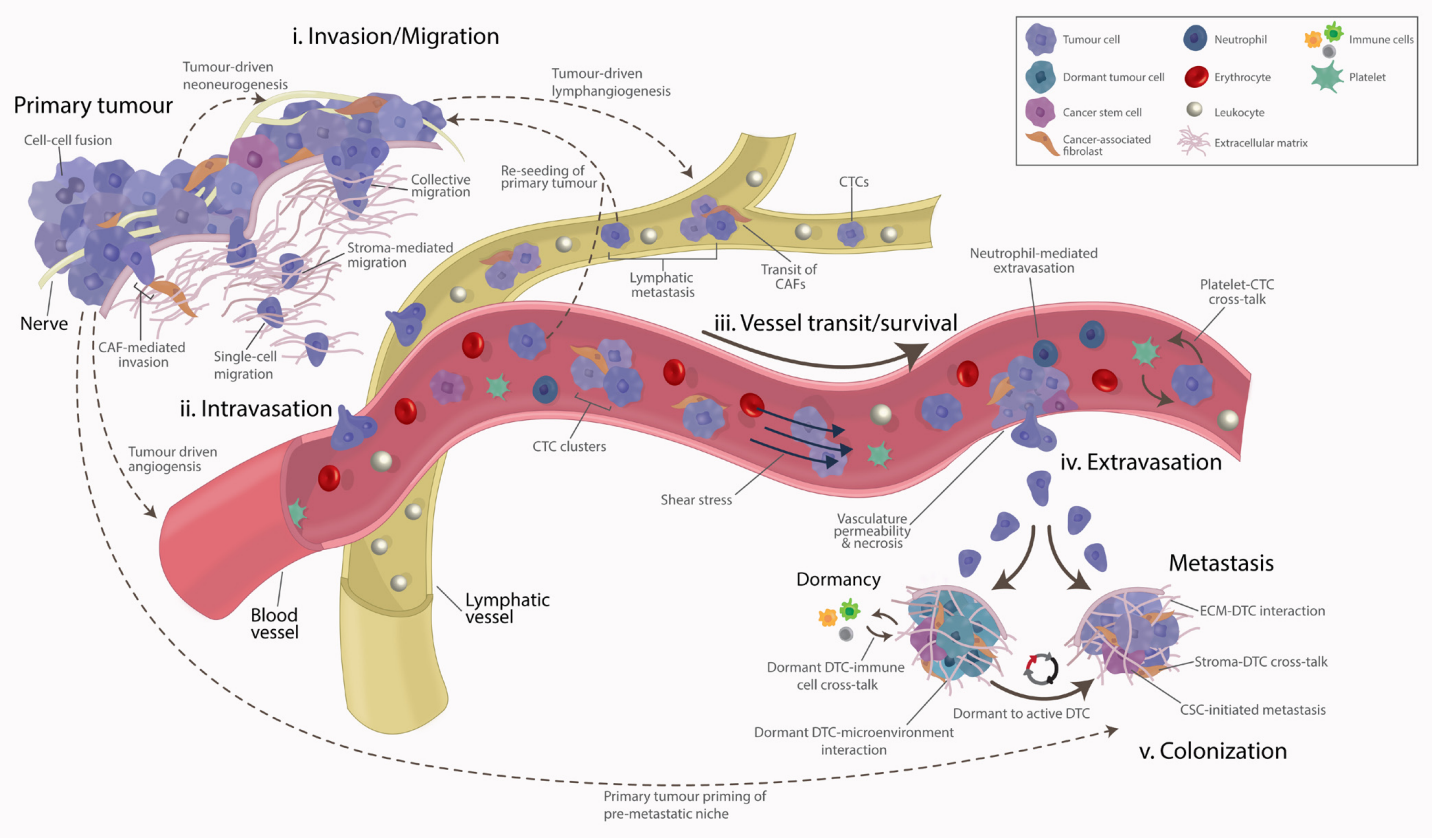
Tumour metastasis is a vast and interconnected array of dynamic and systemic events encompassing both spatial and temporal events. The process can be broadly divided into the following stages: ( **i**) invasion/migration at/near the primary tumour, ( **ii**) intravasation into the local blood and lymphatic vessels, ( **iii**) survival and transit of cancer cells in the circulation/lymphatics, ( **iv**) arrest and extravasation at secondary sites, and ( **v**) overt colonisation of secondary sites.

In almost all solid tumours, the single biggest cause of mortality is metastasis ^1^. Metastasis is the spread of tumour cells away from the primary site of origin and subsequent colonisation of distinct secondary sites ^2^. The process of metastasis and the formation of metastases are inherently inefficient ^3^ yet when successful will typically render the cancer incurable ^1, 4, 5^. Tumour progression to metastasis is not a tumour cell-autonomous program ^6^. It is a multifaceted and complex series of events ^7^, which is influenced at all stages by the intrinsic cellular mutational burden and the numerous bidirectional interactions between malignant and non-malignant cell types and is continuously fine-tuned by the various extrinsic microenvironmental niches, including the biochemistry and biomechanics of the extracellular matrix (ECM) ^8, 9^, and availability and activity of growth factors. This process continually evolves depending on the local and distal microenvironments that tumour cells find themselves within or transiting through ^8, 10, 11^ ( Figure 1) and is tuned by inflammation, angiogenesis, lymphangiogenesis, neoneurogenesis ^12– 14^, and systemic physiologic stress-responsive pathways such as the sympathetic nervous system ^15, 16^. Finally, tumour cells have been known for decades to have the capacity to fuse with one another, leading to further genetic instability, although how this fusion of tumour cells drives the biology of cancer is not yet clear ^17– 19^. As a result, our current understanding of how microenvironmental and macroenvironmental cues intersect with intrinsic cancer cell properties to regulate metastatic dissemination is ever-expanding.

Metastasis as a process is one of the most technically challenging aspects of cancer biology to study ^20– 28^. As a result, new platforms and technologies are continuously being developed to better understand this process ^22, 29^. In this review, we discuss some of the recent advances as well as emerging tools and methodologies being deployed to study metastasis and how the information gleaned is re-shaping our understanding of metastatic dissemination.

## The process of metastasis

Metastases, or metastatic disease, is the end result of a vast and interconnected set of dynamic and systemic events encompassing both spatial and temporal selective pressures exerted upon cancer cells ^30^. Over the past few decades, our understanding of these selective pressures and their importance in the various stages of metastatic dissemination has improved significantly. The entire process of metastasis can be broadly divided into the following stages ( Figure 1):

(i) invasion/migration at/near the primary tumour
(ii) intravasation into the local blood and lymphatic vessels
(iii) survival and transit of cancer cells in the circulation/lymphatics
(iv) arrest and extravasation at secondary sites
(v) overt colonisation of secondary sites

**Figure 2.**
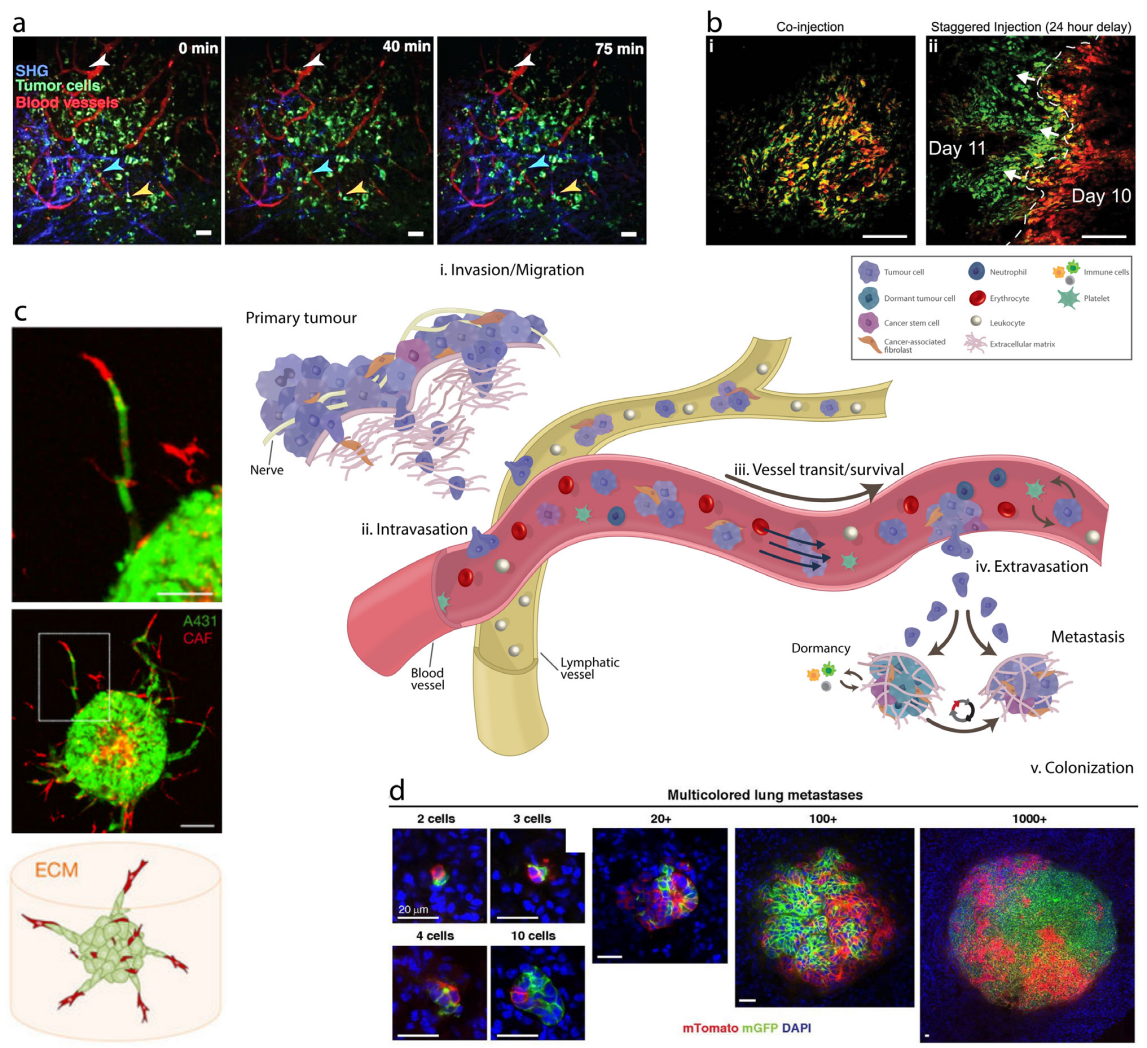
Advances in technologies and tools have allowed us visualise and study some of the stages of metastasis to uncover many of the different mechanisms at play. ( **a**) Time-lapse intravital imaging of cancer cells (green) in association with blood vessels (red) and collagen fibres—blue, detected by second harmonic generation (SHG)—over the course of 75 minutes shows slow movement (arrowheads) of some cancer cells toward blood vessels. From Pereira *et al*. ^69^. Reused with permission from the American Association for the Advancement of Science. ( **b**) Tracking the movement of tumour hypoxia using EF5 and pimonidazole probes. Immunofluorescence of KPC xenograft tumours for EF5 (red) and pimonidazole (green) chemical indicators of tumour hypoxia after either (i) co-injection or (ii) 24-hour delayed administration. Images from Conway *et al*. ^70^, under the terms of the Creative Commons Attribution License (CC BY) ( http://creativecommons.org/licenses/by/4.0/). ( **c**) Confocal images of a spheroid (1:1 mixture of cancer-associated fibroblasts [CAFs] [red] and A431 carcinoma [green] cells) after 60 hours of invasion. CAFs (red) lead collective strands of A431 cells (green). Image, originally published in Labernadie *et al*. ^71^, used with permission from Macmillan Publishers Ltd. ( **d**) Multicellular seeding is a frequent mechanism for distant metastasis. Via Cre recombinase technology, mosaic (red/green) tumour organoids are created and transplanted into non-fluorescent host mice. After 6 to 8 weeks, the lungs of these mice are harvested. Metastases arising exclusively from single-cell seeding produce only single-colour metastases (red OR green). In contrast, multicellular seeding produces metastases with both colours (red AND green). Representative micrographs of polyclonal lung metastases of different sizes from Cheung *et al*. ^36^. Scale bars = ( **a**) 50 μm and ( **b**, **c**) 100 μm.

These different elements ( Figure 1) are often seen as distinct yet interconnected progressive stages of a linear cascade, typically associated with the later stages of primary tumour growth. However, we are now beginning to realise that this is far from accurate. Metastatic dissemination can occur from the earliest point of tumourigenesis, prior to the clinical manifestation of tumours ^31– 33^, and has been shown to be mediated through processes such as ‘delamination’, whereby cancer cells leave the epithelia and cross the basement membrane ^34^. In many patients, metastasis has already occurred by the time of diagnosis, and, as a result, metastasis prevention may be too late ^35^. Nonetheless, developing a deeper understanding of the process of metastasis which leads to overt metastatic disease, along with the attributes that the cells selected by this process possess, will be critical for treating metastatic disease ^4^ and preventing further metastasis in surgically non-resectable patients. Furthermore, the frequent occurrence of multicellular seeding, whereby multiple primary tumour clones come together to form aggressive polyclonal metastases ^36^ ( Figure 2d), and tumour reseeding, whereby circulating tumour cells (CTCs) may return to the primary tumour ^37^, both support the need for continued research into the metastatic process.

Metastasis involves the selection of traits that are advantageous for the survival of cancer cells. Advances in sequencing platforms ^38– 40^ have shown us that micro-evolutionary genetic changes, including somatic mutations, copy number alterations and structural variants in the genome, alongside heritable factors, are detectable independently at both primary and secondary sites as a result of site-specific, context-dependent selective pressures ^6, 33, 41– 43^. This has allowed the identification of hallmark mutational signatures in many different cancer types as well as their metastases ^44– 47^ and is facilitating a deeper subclassification of specific cancers ^48– 52^. In addition, the precise cancer cell of origin has been shown to heavily influence the trajectory of this entire evolutionary process ^53^. Yet, despite these advances, progress has been painfully slow in translating this genetic information into improved clinical outcomes for patients. As such, more effective translational research to assist in contextualising this genetic information against the concomitant recruitment of traits in the tumour stroma and secondary tissues and organs is required yet is not always easy to achieve ^54^. Such research would allow the dissection of the additional layers of complexity at epigenetic, post-transcriptional, and post-translational levels that regulate expression patterns in different tissue microenvironments. Nonetheless, the concept of metastasis as a successive, linear, and discrete stage-centric process, directed solely by the accumulation of genetic mutations, is flawed and has challenged us to re-examine how we both study and effectively target metastasis and metastases ^55^.

The development of new approaches to detect and quantify sparsely distributed metastatic cells throughout the body at early stages in *in vivo* tumour models is underway ^56^. However, in the clinical setting, the current tumour staging procedures and even our highest-resolution imaging technologies are not yet sensitive enough to detect micro-metastases or early tumour cell dissemination, the key events in primary tumour progression to metastasis. Similarly, neither *in vitro* nor *in silico* tools can accurately recapitulate all stages of metastasis, and more holistic approaches using animal models remain the gold standard ^21, 25, 57– 59^. A new era of translational research is developing, and the insights that it brings are rapidly causing paradigm shifts in our understanding of metastatic phenomena.

## Getting things moving: cancer cell migration and invasion

Without question, for metastasis to occur, cancer cells must leave the primary tumour ( Figure 1i). This requires the activation and engagement of cellular mechanisms enabling cell movement, adhesion to or degradation of the ECM (or both), and the weakening of cell–cell adhesions to facilitate dissociation from epithelial neighbours. In particular, this centres around actomyosin contractility, which underpins and drives cell migration and invasion ^60^. Cancer invasion is initiated and maintained by signalling pathways (such as the coordinated activity of the RhoGTPases RhoA, Rac1, and Cdc42 ^61^) that act to control cytoskeletal dynamics in tumour cells and the turnover of cell–ECM and cell–cell junctions to allow cell migration into the adjacent surrounding tissue ( Figure 1i). This process is highly adaptive, being influenced by intrinsic and extrinsic factors, and is typically temporary, having the potential to be reversed. Ultimately, it allows cancer cells to overcome obstacles that would typically impede movement ^62^.

The processes that are activated in cancer cells are similar to those seen in normal cells during embryonic development. These processes allow cancer cells to adapt to their microenvironment and are elicited through changes in cancer cell phenotype and are facilitated, in some situations, by what is known as epithelial-to-mesenchymal transition (EMT) ^63^. The process of EMT is underpinned predominantly by the SNAIL, TWIST, ZEB, and other transcription factor families ^64, 65^. In cancer, EMT is thought to play a role in a cancer cell’s acquisition of a stem-like and motile/migratory phenotype, in part through interaction with other important signalling pathways such as the Hippo pathway ^66^. EMT in cancer, however, is not a one-directional permanent program defined by a single pathway ^63^. Instead, it is a partial or reversible process that depends on the intrinsic and extrinsic stimuli that cancer cells receive. This subtle but critical point is what appears to allow cancer cells to undergo both EMT and reciprocal mesenchymal-to-epithelial transition (known as MET) at different stages and locations of the metastatic process ^67^.

The development of new molecular biology approaches and advanced intravital imaging techniques is providing researchers with novel tools for understanding the importance of EMT in cancer progression and metastasis ^68^. There likely exists both EMT-dependent and EMT-independent mechanisms for metastasis, although as yet the specific contexts for each in different cancer types remain elusive. For example, studies on the reversibility of EMT, and in particular the role of EMT markers such as E-cadherin, have shown that fine-tuned modulation of EMT allows switching between stationary and mobile states, whereas others have shown that EMT may be important in cancer stem cell capacity and sensitivity to chemotherapy ^72– 74^.

Nonetheless, once acquired, cell movement, broadly speaking, occurs in one of two modes: either individual or collective cell migration ^75^ ( Figure 1i). The switch between the two depends heavily on and responds to the physical and molecular triggers present within the microenvironment ^76^. As cancer cells transit within the many different, physiologically distinct, and often hostile multicellular microenvironments, they sense and respond to a plethora of cues, including the biomechanical and biochemical properties of the ECM ^77, 78^. In doing so, cancer cells generate both transient and permanent alterations, including ECM remodelling ^79, 80^, which leads to the co-evolution of both the cancer cells themselves and the tissues through which they transit ^81^. For example, changes in type I collagen organisation are evident in primary and secondary breast cancer sites ^82– 86^, fibronectin levels are altered in ovarian cancer ^87^, and post-translational cross-linking of fibrillar collagens is observed in pancreatic ^88^, breast ^89, 90^, and colorectal ^91, 92^ cancer, all of which are closely linked to disease progression and metastatic dissemination ( Figure 1i).

Interestingly, and perhaps counter-intuitively, the maintenance of epithelial traits during collective cell migration, whereby E-cadherin-dependent cell–cell contacts are maintained, has only recently emerged but has already been shown to be involved in the progression of colorectal cancer ^93^, head and neck squamous cell carcinoma (HNSCC) ^94^, pancreatic cancer ^95^, and breast cancer ^36, 96^. An interesting consideration is whether all migrating cancer cells retain such traits or whether indeed fine-tuning of EMT at a population level by the microenvironment is important ^97^ such that only leading cells at the invasive front retain some crucial epithelial traits and acquire new mesenchymal ones ^98^. Whilst detailed investigation into the underlying mechanisms and generation of new tools ^95, 99– 102^ are underway, it is clear that there is still much we do not know.

Furthermore, in recent years, it has been shown that cancer cell invasion and metastasis are not necessarily cancer cell-autonomous events. Resident stromal cells can be co-opted by cancer cells to facilitate and accelerate processes such as cancer cell invasion. Cancer-associated fibroblasts (CAFs) have been shown to promote cancer cell invasion and metastasis ^103^ through a number of mechanisms, including exerting physical forces on cancer cells via heterotypic E-cadherin/N-cadherin adhesions that enable collective invasion ^71^ ( Figure 1i and Figure 2c). In addition, it is known that CAFs heavily influence cancer cell behaviour by inducing processes such as EMT to initiate their invasion ^104– 107^ or driving apoptosis to facilitate the switch between expansive invasion and CAF-led invasion ^108^. Thus, the concept that tumours behave as communities ^109^, in which cooperative behaviour occurs not only between cancer cell subclones but also between malignant and non-malignant cells ^110^, adds significantly to the layers of complexity in treating these highly heterogeneous tumours. With this in mind, investigators are undertaking mathematical modelling to better understand the dynamics of cell–cell as well as cell–microenvironmental reciprocities that govern metastatic priming and progression ^111– 114^.

A key element that can permit or restrain the invasion of primary tumour cells into the surrounding tissue is the local remodelling of the host microenvironment and in particular the ECM ( Figure 1i). Both normal and tumour-associated ECM is deposited, remodelled, and degraded on a continuous basis. However, the tumour-associated ECM in particular is also associated with altered post-translational modification, such as cross-linking, leading to the generation of a dense and usually stiffer fibrotic microenvironment that is pro-tumourigenic ^11^. For years, despite an expanding body of knowledge to the contrary, it was generally believed that this extensive deposition and remodelling of the tumour ECM merely accompanied tumour growth. More recently, however, it has been widely accepted that ECM remodelling is an active contributor to driving cancer progression ^115^ through clustering of integrins and other receptors, leading to downstream activation of intracellular kinase signalling pathways ^116^, which subsequently alter, among other things, EMT and cancer cell migration and invasion ^117^ ( Figure 1i).

In addition to changes in the ECM at the primary tumour, there are significant changes in the resident non-malignant cell populations recruited to, or excluded from, the tumour and their activation states. Advances in technologies such as intravital imaging have allowed us to uncover mechanisms by which tumour cells manipulate the normal tissues within which they grow in order to facilitate disease progression ^59, 118, 119^. Several recent articles have shown the close interplay among CAFs, primary tumour-associated ECM remodelling, and progression of desmoplastic tumours (those surrounded by dense fibrous tissue) such as pancreatic ductal adenocarcinoma ^88, 120– 122^ and breast cancer ^123^. Most surprising is that CAFs, and the ECM remodelling they underpin, have been shown to play both pro- and anti-tumourigenic roles ^124, 125^, highlighting how simple removal of the stroma may not be a suitable therapeutic approach and showing that, instead, subtler approaches such as stromal re-engineering or normalisation, or short-term ‘priming’ interventions ^120^, may represent a more robust approach ^126, 127^.

Taking this a step further, there have been several recent studies aimed at mapping the changes in the matrisome (the inventory of all ECM constituents) of primary and metastatic lesions ^128^ in order to generate ECM signatures that could be used to predict outcome and metastasis across different tumours ^129– 131^. Because the majority of structural ECM proteins exhibit a remarkable longevity *in vivo*, often measured in weeks and months and even years ^8^, as opposed to hours for intracellular proteins, we argue that the tumour-specific blend of ECM molecules records a history of tumour evolution ^132^. As such, it has the potential to allow us to better understand how a specific tumour has emerged. Furthermore, this longevity appears to be tissue and tumour type specific. The emergence of ECM signatures to stratify patients with cancer is already providing useful predictors of disease staging ^130, 131^, and ECM molecules such as tenascins, periostin, and versicans have been linked to tumour progression and eventually could be used to identify early signs of metastasis ^133– 136^. In addition, just as we currently use genomic signatures to identify high-risk patients and predict outcome across a wide array of cancer types, ECM-based proteomic signatures are emerging, and it will not be long before these can be routinely used in the clinic for patient stratification ^132^.

Yet it is not only cells that move around the tumour. Other non-cellular physiological elements such as hypoxia have recently been shown to move within the three-dimensional tumour using dual PLIM/FLIM (phosphorescence lifetime imaging microscopy/fluorescence lifetime imaging microscopy) intravital imaging ^70^ ( Figure 2b). The importance of this phenomenon is not to be underestimated, since hypoxia is well known to trigger cell invasion and migration as well as other biological effects such as altered response to therapy ^70^. Thus, daily fluctuations in oxygenation status across the tumour likely reshape the microenvironment, both activating and deactivating signalling pathways and gene expression programs in normal and tumour cells, blunting therapy efficacy and thereby having long-term consequences. Furthermore, it was recently shown that, in breast cancer models, intermittent hypoxia, and not chronic hypoxia, actually promotes clonal diversity and enhances metastatic seeding to secondary organs ^137^.

## Going with the flow: intravasation into blood and lymphatics

Intravasation is the active entry of cancer cells into the circulation in order to spread around the body ( Figure 1ii and Figure 2a). Logic dictates that it would follow local invasion away from a tumour toward a vessel, and one of the critical requirements for this would be the ability to activate cellular programs that would act to help tumour cells to transverse the endothelial layer of vessels to enter the bloodstream. However, recent evidence has suggested that intratumoural intravasation does not need to be preceded by local invasion and in fact may proceed in parallel to, or independent of, tumour cell invasion into the surrounding stroma ^138^. Either way, intravasation as a process has been incredibly difficult to visualise and model and so has led researchers to believe it is a rare event. This is in contrast to publications showing that, on average, somewhere in the region of 1 million cancer cells per 1 g of tumour tissue can enter and spread daily within the circulation ^139^. Successful studies to date have shown that the escape of cancer cells from the primary tumour into the circulation can occur as both single cells or clusters of a few to a dozen strands or sheets ^140– 143^ ( Figure 1). What governs the spatial and temporal cues for cancer cell intravasation is still not fully elucidated, but evidence points toward intrinsic cancer cell cues, the activity of stromal cell populations such as macrophages ^110, 144^, and organisation of the ECM. For example, cells may orientate according to ECM structures such as collagen fibres that then can direct tumour cell intravasation in *in vitro* breast cancer cells ^145^.

The vast majority of solid tumours are also able to drive *de novo* angiogenesis (the growth of new blood vessels) ( Figure 1), and malignant progression is typically associated with, and likely even depends on, an angiogenic switch ^146^. Tumour angiogenesis is driven through the secretion of pro-angiogenic growth factors, recruitment of immune cells, and alteration of the perivascular ECM by both tumour cells and associated stromal cells. The generation of leaky tumour vessels is thought to facilitate the dissemination of tumour cells throughout the body and thus represents a viable therapeutic intervention. However, at present, more work is needed to determine whether the majority of intravasation happens predominantly at main vessels or these angiogenic capillary branches. A more comprehensive coverage of angiogenesis in cancer and its therapeutic potential has been reviewed ^146– 148^. However, it must be noted that not all solid tumours form or require new vessels for intravasation to occur, and the existence of non-angiogenic tumours is becoming increasingly recognised ^149^.

In addition to metastatic spread through the circulation, an alternative route of dissemination for cancer cells is through the lymphatics ( Figure 1ii). Lymphatic metastases may be the preferred route of dissemination for some tumour types such as breast cancer ^150^ and rhabdomyosarcoma ^151^, which, compared with other types of solid tumours, show a higher propensity for lymph node metastasis. Indeed, in many tumour types, the extent of lymph node involvement is a crucial prognostic factor for the disease. Recently, it has been shown that during metastasis, cancer cells escape the primary tumour, intravasate into lymphatic vessels, and reach draining sentinel lymph nodes well before they appear to overtly colonise distant organs via the blood circulation ^152^. This process, shown to occur in mammary carcinoma, squamous cell carcinoma, and melanoma model systems, implicates lymph node metastases as a key step for establishing distant metastases of these tumours ^69^. Nonetheless, metastatic disease as a whole is likely to consist of a complex interplay between disseminating cancer cells exiting the tumours via a combination of both routes, modulated by local and systemic factors and possibly even by sex in cases such as renal clear cell carcinoma where androgen receptor (AR) has been shown to increase haematogenous metastasis yet decrease lymphatic metastasis ^153^.

It has been shown that tumours can also promote lymphangiogenesis (the formation of lymphatic vessels) ( Figure 1), which in turn acts to promote cancer cell dissemination ^154^. Aberrant lymphangiogenesis and restructuring of lymphatic networks have been shown to significantly enhance metastasis to both regional lymph nodes and distal organs ^155^ through the secretion of various factors such as vascular endothelial growth factor A/D (VEGF-A/D) ^156^, VEGF-C ^150, 157^, interleukin 1 beta (IL-1β) ^158^, fibroblast growth factor (FGF) ^159^, ECM components such as periostin ^160^, or even chronic stress activation of the sympathetic nervous system ^161^.

Whilst lymph node spread of cancer has been known for decades, more recent evidence has implicated the lymphatics not simply as passive highways for tumour cell spread but also as facilitators in many other processes, including the active recruitment of tumour cells to local and distal lymph nodes ^162^ through mechanisms such as CCL21–CCR7 signalling ^163, 164^, promoting the survival of metastasising cancer stem cells via CXCL12–CXCR4 signalling ^165, 166^, and modulating the host inflammatory response to alter tumour immune surveillance ^167– 170^. Inflammation is a critical component of tumour progression, and inflammatory cells are seen as an indispensable participant in progression. Inflammatory cells have been shown to alter cancer cell proliferation, survival, and migration. In some circumstances, cancer cells have co-opted some of the signalling molecules of the innate immune system, including chemokines and their receptors (such as CXCL12–CXCR4 and CCL21–CCR7 mentioned above), to facilitate invasion, migration, and metastasis. As advances in non-invasive imaging technologies improve and allow us to visualise the lymphatics with greater resolution, and with the development of new tools such as the ‘MetAlert’ mice ^171^, which serve to visualise lymphovascular niches in whole animals, we can begin to study the function of tumour-associated lymphatics on metastatic dissemination as well as during therapeutic response.

## The road to metastasis: circulating tumour cells

In recent years, an increasing body of evidence has been supporting the primary role for CTCs as the major contributor to metastatic relapse in patients with cancer ^172^ ( Figure 1iii). This has fuelled an explosion of interest in their detection and quantification. Indeed, CTCs have been reported in almost all epithelia-derived cancers, including head and neck ^173^, lung ^174^, gastrointestinal (including pancreatic, colorectal and, gastric) ^175– 179^, and breast ^180– 187^ cancer.

With potentially hundreds of thousands of tumour cells intravasating into the bloodstream, it appears that only a small fraction of CTCs are capable of surviving and extravasating into distant sites to persist as disseminated tumour cells (DTCs) ^188^. Thus, in order for CTCs to become DTCs, they face a number of obstacles that they must overcome to survive whilst transiting within the bloodstream ^189^. Studies have shown that CTCs travel either as individual cells or, more often, as clusters ^190^ ( Figure 1iii). These clusters appear in some cases to be heterogeneous in nature, exhibiting combinations of epithelial and mesenchymal traits ^143^. This reintroduces the role of the EMT program in the process of intravasation and cancer cell dissemination. These clusters appear to maintain a partial EMT program which subsequently may facilitate a more robust resistance to apoptosis and an increased propensity to seed and survive at secondary sites ^191^. This resistance to anoikis (apoptosis induced by inadequate or inappropriate cell–cell or cell–ECM interactions) in CTCs has been shown to be driven through various mechanisms, including expression of the tyrosine kinase receptor TrkB ^192^ or activation of non-canonical Wnt signalling ^193^.

Transit within the circulatory system represents one of the most vulnerable times for disseminating cancer cells, and the importance of cooperative host–tumour cell interactions during this time should not be underestimated ^194^ ( Figure 1iii). During transit, there is significant cross-talk among tumour cells, accompanying CAFs, platelets, leukocytes, and endothelial cells. These cell–cell contacts and paracrine cell–cell interactions occur both temporally and spatially during transit and at sites of extravasation. For example, CTCs have been shown to associate with activated platelets, which secrete protective signals, such as transforming growth factor-beta (TGF-β), which in turn upregulates nuclear factor kappa B (NFκB) signalling in CTCs, potentially substituting for stromal interactions found at primary and secondary sites ^195^. Furthermore, these platelets have been shown to form protective shields via the deposition of fibrinogen ^196^ and tissue factor (TF) ^197^. In some cases, it has been shown that disseminating tumour cells carry with them primary tumour CAFs along with stromal ECM components ^198^, which subsequently act to facilitate seeding and overt colonisation at secondary sites.

Tools and technologies for the detection of CTCs in the peripheral blood are continuously evolving, yet none has reached the ‘gold’ standard of sensitivity and, more importantly, of specificity ^172^. Nonetheless, every one of these studies supports a critical role for CTCs in metastatic dissemination. To that end, it is now widely accepted that targeting CTCs during haematogenous transport within the circulation may offer an effective approach to targeting the metastatic process, which could lead to the reduction of cancer morbidity and mortality in early stage cancer patients without already-established metastatic diease ^199^.

Of note, one of the major forces that CTCs experience during transit in the circulation is shear stress. The shearing forces exerted on CTCs are caused by the movement of blood over the cell surface. It is heavily influenced by both the viscosity and the velocity of the blood flow ^200^. It is perhaps not surprising that tumour cells have been shown to be more resistant to haemodynamic shear stress than normal cells ^201, 202^ and that this feature is crucial not only to survival in the bloodstream but also to the activation of mechanotransduction signalling during attachment at and extravasation into secondary sites ^203^. Furthermore, the generation of tumour microparticles from CTCs attached to vessel walls as a result of shear flow in capillaries within the lung vasculature has been shown to modulate local immune cell behaviour and confer anti-metastatic protection at metastatic sites ^204^.

## Next stop, please! Tumour cell extravasation

For decades, it was thought that the specific patterns of metastatic dissemination observed in patients could be explained solely by the dynamics of haematogenous flow ^7^. Not until the 1970s was it demonstrated that regardless of the importance of blood flow, successful metastatic colonisation could occur only at certain organ sites ^205, 206^. These studies were the first to provide experimental evidence for organotropic metastasis. Since then, several studies have dissected the various elements of CTC attachment to and extravasation at secondary sites of metastasis. These studies have uncovered critical elements, such as the ability of CTC clusters to manoeuvre through capillary-sized vessels, doing so as a single-cell chain held together through adhesive interactions ^207^. Another study demonstrated that CTC induction of ATP secretion from accompanying activated platelets is able to render the vasculature more permeable by acting on P2Y2 receptors expressed by endothelial cells ^208^. Similarly, CTC-driven platelet-induced alpha-granule secretion contains a wide range of metastasis-promoting growth factors and cytokines that support cancer cell extravasation and survival at secondary sites ^195^.

A recently discovered phenomenon, which has also been shown to play a role in metastasis of some solid tumours, is the production of neutrophil extracellular traps (NETs) ^209^. NETs are extracellular DNA structures that are typically ‘cast’ by neutrophils in response to infection. However, it has been shown that some metastatic cancer cells can stimulate neutrophils to form NETs, which ultimately act to support metastatic colonisation of secondary sites, and these NETs have been observed in both *in vivo* models and clinical samples ^210^.

Other work has shown that CTC secretion of the CCL2 chemokine is capable of directly inducing vascular permeability ^211^ and subsequently recruiting pro-tumourigenic CCR2 receptor-positive inflammatory monocytes to sites of extravasation ^212^. In addition, the presence of distinct and specific ‘tumour microenvironment of metastasis’ (so-called TMEM in models of breast cancer dissemination) has been described in both genetically engineered models of breast cancer and human breast cancer patients. It has been shown, using intravital microscopy, that a local loss of vascular junctions at TMEMs, mediated by TIE2 ^high^ macrophage-derived VEGF-A, facilitates cancer cell intravasation and metastasis ^213^ ( Figure 1v). Additional factors have been implicated in altering vascular permeability, secreted either locally by tumour cells within the vasculature or systemically from the primary tumour ^214^, to facilitate the alteration of vascular endothelial barriers, including microRNAs (miRs) ^215^, secreted factors such as VEGF, a disintegrin and metalloproteinase domain-containing protein 12 (ADAM12), epiregulin, cyclooxygenase-2, matrix metalloproteinase-1 (MMP-1) and MMP-2 ^216^, angiopoietin-like 4 ^217^, angiotensin II (ANG-2), MMP-3 and MMP-10 ^218^, and finally stromal cell-derived factor 1 (SDF1) ^219^. Many of these factors have also been implicated in the generation of pre-metastatic niches (reviewed elsewhere ^220^). Interestingly, a novel mechanism has recently been described in which CTCs, once arrested on the endothelial wall of blood vessels, can extravasate and coordinate the formation of overt lung metastases via the induction of programmed necrosis (necroptosis) in endothelial cells of vessel walls ^221^. Neutrophils also appear to play an important role in regulating the survival and extravasation of CTCs from the bloodstream, through direct interaction ^222, 223^, regulation of natural killer (NK) cell activity via secretion of IL1β or MMPs ^224^ (or both), or altering cytotoxic CD8 ^+^ T-cell responses ^225^. Finally, only very recently, researchers have shown that haemodynamic forces and the speed of circulatory flow alone may be critical components of tuning the arrest, adhesion, and extravasation of CTCs from the circulation ^226^ ( Figure 1v).

## Building new homes: metastatic seeding and tissue colonisation

Despite estimates that over 1 million cancer cells per 1 g of tumour tissue enter the bloodstream daily ^139^, only a very small proportion survive, escape, and become DTCs. An even smaller fraction of these DTCs (that do not become dormant, as discussed in the next section) are capable of progressing toward overt metastases ^188^. It is known that the DTC microenvironment plays an important role in sustaining their survival, regulating their growth, and conferring resistance to therapy ^227^. The ‘seed and soil’ hypothesis proposed by Stephen Paget in 1889 broadly states that colonisation of a secondary site is, in part, dependent on the interactions between tumour cells and the secondary host tissue. That is, inadequate support or cues from secondary tissues, mediated by local resident and recruited cells as well as the ECM, significantly contribute to the inefficiency of the metastatic process.

There is still much discussion as to whether the ability of a tumour cell to overtly colonise a secondary organ is pre-programmed at the primary site prior to leaving or educated upon extravasation at these secondary sites or, more likely, a combination of the two. There is a large body of work addressing how the establishment of pre-metastatic niches and primary tumour-driven remodelling of sites of future metastasis ^53, 228^ cross this divide. Work in the PyMT model of breast carcinogenesis has shown that a rare population of primary tumour-derived cancer stem cells can initiate metastases in the lung and that, accordingly, the ability of these tumours to metastasise is dependent on the induction of periostin expression in secondary sites in order to maintain cancer cell stemness ^229^. Furthermore, the oxygen-rich environment in the lung may act to restrain T-cell responses to extravasating cancer cells and induce tolerance to provide a more hospitable environment for metastatic colonisation ^230^. Similarly, the ability of DTCs to physically interact with the ECM, at least in the context of the lung, appears to be contingent upon their ability to form filopodium-like protrusions that are rich in integrin beta-1 ^231^. DTCs that are unable to sense or respond to these secondary organ cues thus fail to activate the proliferative programs, driven primarily by FAK, SRC, and ERK signalling, that are necessary for overt metastatic colonisation ^92, 232^. As such, it has been shown that targeting Src and ERK signalling pathways may be a potential therapeutic approach to block overt metastatic colonisation of the lung by breast cancer cells ^233^.

Overt colonisation of tissues likely requires a series of tissue-specific events, which may explain the propensity of certain tumours for metastatic organotropism. For example, in the brain, DTCs encounter reactive astrocytes that produce plasminogen activator, which leads to the production of plasmin and induces DTC death. The ability of DTCs to survive in this hostile environment is therefore dependent upon the ability of the cancer cell to express serpins ^234^, which typically are produced by neurons and protect against plasminogen activator-mediated cell death ^235^. Conversely, serpins have also been shown to be important in stromal remodelling and local invasion at the primary tumour in pancreatic cancer ^236^, highlighting tissue- and context-dependent roles for this family across multiple stages of metastatic dissemination. Thus, the ability of DTCs to acquire or express markers of non-malignant resident cells in tissues, and in doing so mimicking these cells, could be a malignant adaptation required for survival and overt secondary organ colonisation. In another example, a study has shown that metastasising breast cancer cells arriving in the brain display a GABAergic phenotype similar to that of neuronal cells, which enhances their survival and subsequent metastatic colonisation ^237^.

In addition to cell-intrinsic properties of the arriving cancer cells, their ability to subvert resident stromal cells to initiate remodelling programs in these new and distinct environments, such as the bone ^238^, is critical in facilitating overt colonisation. Thus, the local stroma, comprising ECM, non-malignant cells, and the signalling molecules they produce, is an integral and vital component of secondary niches that, together with the underlying genetic aberrations in the cancer cells, determines the growth characteristics, morphology, and aggressiveness of disseminating tumour cells ^239^. For example, lung colonisation by breast cancer cells is enhanced by the deposition of the ECM components tenascin C ^133^ and periostin ^229^ or post-translational cross-linking of collagens ^86^.

It is now well established that sites of future metastasis within secondary organs are not merely the passive receivers of CTCs but instead are selectively and actively modified by the primary tumour prior to the arrival of CTCs ^220^. The term pre-metastatic niche was coined over a decade ago to describe the systemic modification of secondary tissue microenvironments to facilitate subsequent metastatic colonisation by disseminating tumour cells ^240^. In order to maximise the chance of overt metastatic colonisation of secondary organs, the combined action of tumour-secreted factors and tumour-shed extracellular vesicles (cargo-containing vesicles that are secreted by cells into the extracellular space and can bind to and be incorporated into other target cells to facilitate cell–cell communication) is required to facilitate this pre-metastatic niche formation. Together, their coordinated action induces changes such as the induction of vascular leakiness ^241^, remodelling of stroma and ECM ^90, 242^, along with systemic effects on the immune system. Many of these secreted factors are transported within cancer exosomes (extracellular vesicles, typically 40 to 100 nm, shed from the surface of cells) possessing unique surface marker compositions, which act to facilitate guiding of the exosomes and their cargo to specific secondary organs of future metastasis ^243– 246^. For example, pancreatic cancer cell-secreted exosomes have been shown to accumulate in secondary tissues such as the liver and lead to the generation of pre-metastatic niches through activating hepatic stellate cells and Kupffer cells to drive ECM remodelling ^243^ and can be detected in the circulating blood, offering promise of potential biomarker applications. Given the technical limitations of studying these early pre-metastatic events *in vivo* and in the clinic, there has recently been a push to develop engineered niche-mimicking biomaterials to better study this process ^247, 248^.

## Lying low: disseminated tumour cell dormancy

Dormancy is defined as the latent state in which (tumour) cells remain quiescent and are reversibly arrested in the G _0_ phase of the cell cycle ^188^. When tumour cells enter a patient’s bloodstream, the cells transit to and lodge in various microenvironments such as niches in the lung tissue or bone marrow. Upon arrival, the tumour cells may become dormant. These dormant tumour cells can spend months, years, and even decades in these niches, which act as a safe haven, in many cases providing protection from adjuvant therapies ^28^. Dormant tumour cells are typically seen as chemotherapy-resistant because they are not actively dividing; however, the molecular mechanisms underlying this resistance are still poorly understood ^227^. There are also emerging arguments in the field that, rather than wait for these metastases to emerge before initiating treatments, it may be more effective to target the dormant metastatic seeds or their dormancy-inducing niches before they re-awaken (or both) ^227^ or, perhaps more controversially, actively stimulate their re-awakening during adjuvant therapy.

In some situations, studies of metastatic tumour dissemination have shown that primary tumour-driven mechanisms act to counter the overt colonisation of secondary tissues and thereby induce dormancy. For example, rather than forming pre-metastatic niches (discussed above) that act to increase the efficiency of metastatic colonisation, tumours may create specialised microenvironments in which tumour cells can become quiescent, allowing DTCs to survive in a dormant state. These ‘sleepy niches’ or ‘silent’ pre-metastatic niches ^220^ result in the extensive delay in the development of overt metastasis. DTCs thus appear to be able to persist long term within organs, re-awakening many months or years later when the host organs inevitably succumb to overt colonisation ^249^. Of note, in addition, there is experimental evidence to show that DTCs can persist in other organs that rarely develop metastases ^250^. However, what governs the re-awakening of dormant DTCs is still the topic of much debate.

An important factor determining the persistence of dormant DTCs appears to be their ability to escape the body’s immune surveillance. Previous work has shown that DTCs can evade NK cell clearance by decreasing the expression of NK ligands, a program that appears to be tightly coupled with their entry into a quiescent state ^251^. Similarly, it appears that, in some cases, DTCs can be held in a state of dormancy by CD4 ^+^ and CD8 ^+^ T cells ^252– 254^. There are also several factors that have been shown to induce or sustain (or both) the dormancy of DTCs in secondary tissues and they tend to be organ specific. For example, bone morphogenetic protein 4 (BMP4) is present in many tissues, yet elevated levels in the lung contribute to modulating prostate ^255^ and breast cancer ^256^ cell dormancy. Breast cancer cells lodged within the bone marrow can activate Src signalling and expression of the CXCR4 receptor which in turn activates pro-survival signalling in response to bone-derived CXCL12 ^257^. Similarly, in the bone marrow, secreted factors such as BMP7 and TGF-β2, as well as ECM components such as secreted protein acidic and rich in cysteine (SPARC), have been shown to modulate HNSCC ^258^ and prostate cancer ^259, 260^ cell dormancy. Thrombospondin-1 (TSP1) produced from mature endothelial cells and deposited into the microvascular basement membrane is able to confine DTCs to a quiescent state in some tissues ^261^. However, given the ubiquitous nature of TSP1 in other tissues, it strongly indicates that a co-operative interaction with other factors present within each tissue-specific context may be at play. In this particular study, the authors elegantly dissect the role of vascular niches, demonstrating that TSP1 suppresses DTC outgrowth in both the lung and the bone marrow but not in the brain ^261^.

Both inflammation and ECM remodelling programs elicit profound effects on cell behaviour, including DTCs and cellular dormancy programs. The outgrowth of previously dormant DTCs in the lung has been shown to be activated by both inflammation ^262^ and TGF-β-driven fibrotic type I collagen remodelling ^263^. Similarly, other tissue-resident cells, including osteoblasts and osteoclasts, have been shown to control the switching of dormancy programs within the endosteal niche in multiple myeloma ^264^.

## Clinical translation and implications

Clinically, metastatic disease represents a major challenge and is responsible for more than 90% of deaths associated with solid tumours ^265^. Conventional drugs for cancer treatment are largely cytostatic drugs aimed at targeting intrinsic cancer cell mechanisms such as cell cycle progression. Although in many instances they are successful in reducing the size of primary tumours, they have been shown to have little effect on DTCs, potentially owing to the increased heterogeneity and significant mutational burden of DTCs, which facilitates efficient evasion of cell death ^1, 265, 266^. Furthermore, evidence suggests that some chemotherapies may trigger metastasis through increasing intravasation ^267^. Thus, much research has turned to finding drugs which interfere with cell motility, targeting phases such as cancer cell invasion and migration through the surrounding ECM ^265^, intravasation, and extravasation.

Treating cancer metastasis is further challenged by the logistical and indeed ethical difficulties in evaluating metastasis formation and development in clinical trials. Running metastasis-preventing trials on patients with early stage cancer using survival and reduction of metastases as the endpoint is not always viable, as these studies will be lengthy and will require a large number of patients with otherwise relatively good survival prospects. One must also remember that the metastatic pathway is a dynamic, ongoing process, which has, in many patients, already occurred before primary diagnosis, meaning that successful treatment would require targeting of early or already-established metastasis rather than the initial process of dissemination and colonisation. Similarly, it is highly unlikely that a single metastasis-preventing agent will be maximally effective, and so co-targeting multiple elements of the metastatic process, coupled with new clinical trial designs, is required, though not always readily achievable, to improve patient outcome and improve survival. However, given the current landscape, there is still much work needed before successful targeting of established metastasis can become a clinical reality.
